# Robust Stereo Visual Inertial Navigation System Based on Multi-Stage Outlier Removal in Dynamic Environments

**DOI:** 10.3390/s20102922

**Published:** 2020-05-21

**Authors:** Dinh Van Nam, Kim Gon-Woo

**Affiliations:** Intelligent Robotics Laboratory, Department of Control and Robot Engineering, Chungbuk National University, Chungdae-ro 1, Seowon-Gu, Cheongju-si 28644, Chungbuk, Korea; quangnam.auto.tech@gmail.com

**Keywords:** vision aided-inertial navigation system, visual-inertial odometry, visual SLAM, extended kalman filter, bayes filtering

## Abstract

Robotic mapping and odometry are the primary competencies of a navigation system for an autonomous mobile robot. However, the state estimation of the robot typically mixes with a drift over time, and its accuracy is degraded critically when using only proprioceptive sensors in indoor environments. Besides, the accuracy of an ego-motion estimated state is severely diminished in dynamic environments because of the influences of both the dynamic objects and light reflection. To this end, the multi-sensor fusion technique is employed to bound the navigation error by adopting the complementary nature of the Inertial Measurement Unit (IMU) and the bearing information of the camera. In this paper, we propose a robust tightly-coupled Visual-Inertial Navigation System (VINS) based on multi-stage outlier removal using the Multi-State Constraint Kalman Filter (MSCKF) framework. First, an efficient and lightweight VINS algorithm is developed for the robust state estimation of a mobile robot by practicing a stereo camera and an IMU towards dynamic indoor environments. Furthermore, we propose strategies to deal with the impacts of dynamic objects by using multi-stage outlier removal based on the feedback information of estimated states. The proposed VINS is implemented and validated through public datasets. In addition, we develop a sensor system and evaluate the VINS algorithm in the dynamic indoor environment with different scenarios. The experimental results show better performance in terms of robustness and accuracy with low computation complexity as compared to state-of-the-art approaches.

## 1. Introduction

In recent years, the field of robotics has witnessed remarkable advances in both academia and the industry with the assistance of Artificial Intelligence (AI). The evolution of technology has awakened various real-time applications of the mobile robot, such as search and rescue missions in disasters, ship and deliver small packages, and self-driving vehicles [1,2]. The localization system of an autonomous mobile robot is a crucial competence. However, the system typically involves an accumulation error over time, particularly in long-term navigation. Although Real-Time Kinematic (RTK)-GPS can provide outstanding precision for outdoor localization, it is not suitable for indoor or GPS-denied outdoor environments such as under bridges or high buildings. To this end, the Multi-Sensor Fusion Technique (MSFT) is developed to bound the navigation error by joining the advantages of proprioceptive and exteroceptive sensors. Simultaneous Localization and Mapping (SLAM) has employed MSFT to estimate the state, reconstruct, and understand the surroundings over the last 20 years [3]. It also enables loop closure detection to enhance not only the accuracy but also the certainty of the estimated robot pose for long term navigation. The SLAM system for the mobile robot still needs to improve robustness and performance while exploiting the real-time semantic environment. Recently, Modern Micro-Electro-Mechanical Systems (MEMS) technology has reinforced the abilities of inertial measurement sensors [4,5] with low-cost and low power consumption. The outputs of an Inertial Measurement Unit (IMU) are 3D acceleration and 3D angular velocity. However, the measurements regularly carry the bias system error and random noise. Exteroceptive sensors such as cameras or LiDARs are fused, which can aid inertial sensors to avoid such problems. Nevertheless, LiDAR technology, compared to the camera, is more expensive. Thus, the Visual-Inertial Navigation System (VINS) is developed to complement measurement information from the camera and IMU, which mimics humans [5] and can also achieve high performance in term of accuracy with minimum aided computational cost [4]. The monocular, stereo, 360-degree Omni-directional camera can be fused with inertial data. However, the stereo camera supports depth data and provides more reliable feature tracking. Notably, resource-constrained devices typically are not suitable to utilize a 360–degree Omnidirectional camera. For that reason, the Stereo Visual-Inertial Sensors (Stereo-VINS) are selected to design the localization system.

Numerous VINS algorithms have been provided to the community research, including filtering-based, optimization-based, and deep learning-based. The filtering-based approach is a method that uses Bayes filtering [6], such as the extended Kalman filter and the unscented Kalman filter, which are employed to estimate the most recent robot state [7,8,9,10,11]. In contrast, the optimization-based technique estimates robot states online or offline processing with the graph optimization [12]. The optimization-based VINS can be classified into fixed-lag smoothing and the full smoothing. The fixed-lag smoothing [13,14,15,16] employs the estimating states within a window while concurrently removing the oldest state. The full smoothing algorithm [17,18,19] manipulates the whole history states of the travel pathway with their measurement constraints, then solves extensive nonlinear optimization in terms of the factor graph algorithm [12]. The optimization-based strategy is commonly more reliable than filtering-based and is more robust to outliers. Most recently, deep learning based-VINS [20,21,22] have employed the Convolutional Neural Networks (CNNs) and Long Short Term Memory (LSTM) networks [23] architecture into an end-to-end learning process [24] to handle vision and inertial data simultaneously. The evaluations are sufficient but less precise compared to the model-based systems. Besides, VINS can generally be categorized into the tightly-coupled and loosely-coupled system. The loosely-coupled method individually utilizes its process and then joins the results in the back-end system. The tightly-coupled solution instantly joins all measurement data within a single estimation process, then solves a large sparse optimization problem to achieve more efficiency [25,26]. The Multi-State Constraint Kalman Filter (MSCKF) algorithm, which is an enhancement approach of the filtering-based strategy in terms of the tightly-coupled, marginalizes all visual features state out of the state vector to reduce the computational complexity. The MSCKF problem is well documented in papers [7,8]. Nevertheless, the main drawback of MSCKF is that its process is delayed until all visual-landmarks are obtained. Therefore, the MSCKF cannot manipulate the whole features concurrently at the correction phase. The objective of this work is to develop a robust VINS for resource-constrained devices in real-time applications. Therefore, the MSCKF framework [7] is leveraged with online spatial and temporal calibration [27,28] to reduce the incorrect projection of 3D information and to support better feature matching.

**Figure 1:**
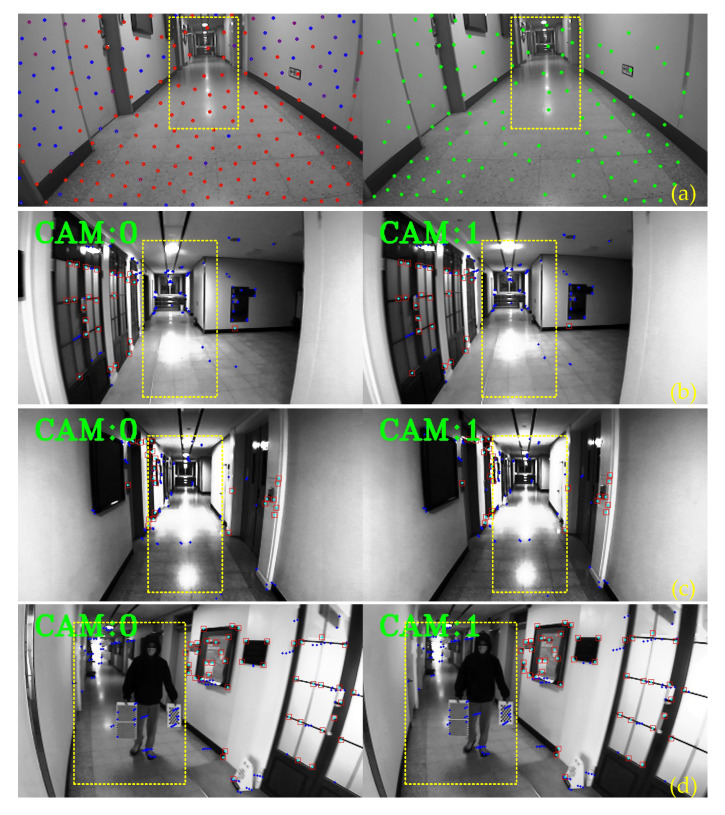
Experimental results in a dynamic environment influenced by illumination and a dynamic object. Herein, yellow rectangles present affected areas, small red squares indicate robust features. (**a**) the VINS-fusion [13], and (**b**–**d**) show the proposed method.

In reality, the ego-estimate accuracy of the Stereo-VINS is still influenced by dynamic objects and the illumination of the surroundings. The incorrect data can be considered as the visual uncertainty information affecting the estimation system immediately. Besides, the limited number of studies focus on how to remove outliers in terms of Vision-Aided Inertial Odometry/SLAM (VIO/VI-SLAM). Most of the studies concentrate on Visual Odometry/SLAM (VO/VSLAM). Typically, outlier removal methods are based on an optical flow technique and can be divided into two categories: One only considers the visual feature position and the other examines both the visual feature and the movements of the camera. A. Amit et al. [29] suggested an optical flow vector with rotation information in one image to find a similar direction, then dismiss outliers by using the most frequent angle vector. However, this study uses a constant threshold to separate inliers and outliers, which is not a robust approach. In [30], Santana Pedro et al. proposed a solution to reduce the influence of rotational movement by separating the obtained image into a half left and a half right, and then to reject outliers efficiently. M. Buczko and V. Willert [31] proposed a study based on the comparison of the optical flow and the camera movement. The error is then applied as a standard to treat rotation errors in the camera motion. Researchers in [32] applied optimization strategies to efficiently classify outliers in the VO problem by using only a monocular camera without the depth information. Nevertheless, the mentioned techniques do not use the information such as the velocity, rotation angle in the sliding window to update the threshold. This work proposes a robust multi-stage outlier removal strategy that leverages all estimated states in the sliding window of VIO to eliminate the visual uncertainty features in the stochastic environment. Figure 1 shows that our proposed method can extract more robust features than a state-of-the-art VINS-fusion [13] influenced by illumination and a dynamic object.

**Figure 2:**
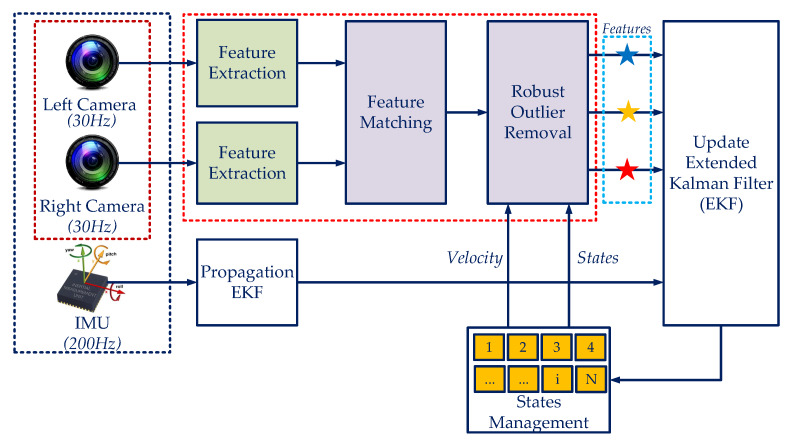
The proposed navigation system architecture is based on a robust visual-inertial fusion with a multi-stage outlier removal. Herein, the features are classified into three classes which are represented by blue, yellow, and red.

In this paper, a robust stereo visual-inertial navigation system for a mobile robot is proposed in dynamic indoor environments. A system architecture is briefly illustrated as in Figure 2, where the inputs are images from the left and right camera and the inertial data from an IMU. Firstly, the feature extraction is utilized to obtain the Features from Accelerated Segment Test (FAST) features [33] from the images.Then, the Kanade–Lucas–Tomasi (KLT)-feature tracker with the RANdom SAmple Consensus (RANSAC) [34] is utilized to match the features. A robust outlier removal method is developed by employing the forward-feedback-up-down tracking, then rejoins the state feedback information of the navigation system to remove the outliers. Once we have robust visual features, then one of Bayes filtering [6] is applied to correct the robot state obtained from the propagation phase. The experimental results with public datasets show that the proposed methodology achieves more reliable performance in both computational complexity and accuracy than state-of-the-art approaches. Besides, the tests are conducted in various scenarios under dynamic environments with an indigenous built sensor setup.

In summary, the key contributions of this work can be summarized as follows:

- We provide the design of a robust stereo vision aided-inertial navigation system with high accuracy and low computational cost based on multi-stage outlier removal towards resource-constrained devices for stochastic environments;
- Multi-stage outlier removal strategies are introduced, in particular an approach with a multi-stage that removes outlier features caused by the influences of the dynamic objects based on the state feedback information in the sliding window is proposed;
- The experimental evaluation of the proposed VINS algorithm is carried out on both public and our datasets for the indoor environment. We demonstrate on relevant datasets that the proposed solution can robustly reject outliers and improve the system’s accuracy both for the static and dynamic environment.

The remainder of this paper is structured as follows, Section 2 introduces the proposed system. In Section 3, we present the strategies to reject outliers. Experimental results are illustrated in Section 4, followed by a conclusion in Section 5.

## 2. Robust Stereo Vision-Aided Inertial Navigation System Estimator Description

**Figure 3:**
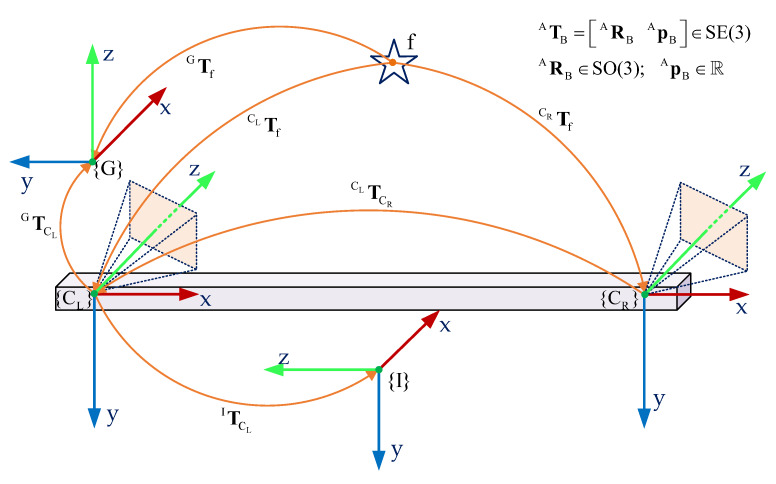
The transformations between the global frame {G}, Inertial Measurement Unit (IMU) frame {I}, right camera frame $\left\{C_{R}\right\}$, and left camera reference frame $\left\{C_{L}\right\}$. The IMU frame {I} is the navigation coordinate. f illustrates the position of a visual feature. ${}^{A}T_{B}=\left[{}^{A}R_{B}\;\;\;{}^{A}p_{B}\right]$ represents the homogeneous transformation from frame B to frame A on SE(3), where R is the rotation matrix on SO(3) and p is the translation.

In this section, we provide the design of a robust stereo vision-aided inertial navigation system. In order to design the system, we first set up a sensor coordinate configuration that consists of the left camera $\left\{C_{L}\right\}$, right camera $\left\{C_{R}\right\}$, and IMU {I} frame as shown in Figure 3. The robot frame is selectively aligned with the inertial frame {I}, determined as a reference frame for the navigation system. The global frame {G} is aligned with the world-centric and is considered as an initial pose of the robot during the operation.

### 2.1. The Definition of System State on Manifolds

Following the MSCKF approach [7,27], we define a state vector of the VINS system at each camera clock instance, which consists of the IMU state concerning the global frame {G} and a series of K camera poses clones in a sliding window. Besides, a group of ρ robust visual landmarks, a set of stereo camera’s extrinsic and intrinsic calibration parameters, and a time offset are also employed. All mentioned information are combined in a unique state vector $x_{\ell }$ represented as:

(1) $$x_{\ell }={\left[x_{I}^{T}\;\;\;\;\;o_{C}^{T}\;\;\;\;\;o_{S}^{T}\;\;\;\;\;o_{Z}^{T}\;\;\;\;\;{}^{C}t_{I}\right]}^{T},$$

where $x_{I}$ as the IMU states can be expressed as:

(2) $$x_{I}={\left[{}^{I_{\ell }}{\bar{q}}_{G}^{T}\;\;\;\;\;{}^{G}p_{I_{\ell }}^{T}\;\;\;\;\;{}^{G}v_{I_{\ell }}^{T}\;\;\;\;\;b_{\omega_{\ell }}^{T}\;\;\;\;\;b_{a_{\ell }}^{T}\right]}^{T}.$$

The Jet Propulsion Laboratory (JPL) unit quaternion ${}^{I}{\bar{q}}_{G}$ in Equation (2) describes a rotation from {G} frame to {I} frame, and ${}^{G}p_{I_{\ell }}$ and ${}^{G}v_{I_{\ell }}$ are the transition and velocity from the IMU {I} frame to global {G} frame at time step *ℓ*, respectively. $b_{\omega }$ and $b_{a}$ are the gyroscope and acceleration biases, sequentially. The inertial vector $x_{I}$ has a total of 15 Degrees of Freedom (DOF) and represented on manifold M with a vector space $R^{12}$ product with unit quaternion space U ($M=U\times R^{12}$). The camera clones collected at different time step within the sliding window are given by:

(3) $$o_{C}={\left[{}^{I_{\ell -1}}x_{C}^{T}\ldots \;\;\;{}^{I_{\ell -i}}x_{C}^{T}\ldots \;\;\;{}^{I_{\ell -K}}x_{C}^{T}\;\;\right]}^{T},$$

where ${}^{I_{\ell -i}}x_{C}^{T}={\left[{}^{I_{\ell -i}}{\bar{q}}_{G}^{T}\;\;\;\;\;{}^{G}p_{I_{\ell -i}}^{T}\right]}^{T}$ is the position of a camera pose clone with respect to the global frame {G} at time instance ℓ−i. The vector $o_{S}$ represents a set of robust visual features ${}^{G}p_{S_{\rho }}$ and is given by:

(4) $$o_{S}={\left[{}^{G}p_{S_{1}}^{T}\ldots \;\;\;\;\;{}^{G}p_{S_{2}}^{T}\;\;\;\ldots \;\;\;\;\;{}^{G}p_{S_{\rho }}^{T}\right]}^{T}.$$

**Figure 4:**
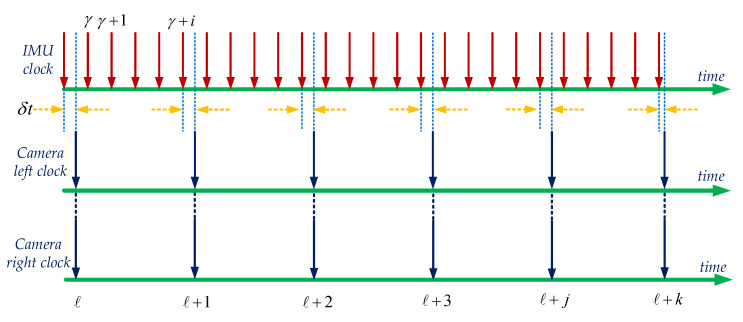
The demonstration of stereo camera and IMU clock. The left and the right camera are synchronized by software while having a time offset between the camera clock and the closest IMU clock.

The calibration vector $o_{Z}$ including extrinsic parameters with rotation ${}^{I}{\bar{q}}_{C_{z}}$ and transition ${}^{C_{z}}p_{I}$ from the IMU frame to the left camera frame, and intrinsic parameters $\vartheta_{z}$ of the stereo camera is expressed by:

(5) $$\begin{array}{c} o_{Z}={\left[{}^{I}x_{Z_{1}}^{T}\ldots \;\;\;\;\;{}^{I}x_{Z_{i}}^{T}\ldots \;\;\;\;\;{}^{I}x_{Z_{z}}^{T}\right]}^{T}, \end{array}$$

where ${}^{I}x_{Z_{i}}^{T}={\left[{}^{I}{\bar{q}}_{C_{i}}^{T}\;\;\;\;\;{}^{C_{i}}p_{I}^{T}\;\;\;\;\;\vartheta_{i}^{T}\right]}^{T}$. Assume that the left and right camera clock are synchronized and have a time offset ${}^{C}t_{I}$ denoted by δt to the IMU clock as shown in Figure 4.

Herein, a unit quaternion $\bar{q}$ can be updated by multiplying an estimated quaternion $\hat{\bar{q}}$ with an error quaternion $\delta \bar{q}$ as yielded $\bar{q}=\delta \bar{q}⊗\hat{\bar{q}}$. A small angle can be appropriated as $\delta \bar{q}≃{\left[\frac{1}{2}\delta \theta^{T}\;\;\;\;\;1\right]}^{T}$ and $R\left(\delta \bar{q}\right)=I_{3\times 3}-\left[\delta \theta_{\times }\right],$ where ⊗ presents the quaternion multiplication, R(.) denotes a 3×3 rotation matrix on the special orthogonal group SO(3) [6,35]. On differentiable manifolds, the *boxplus* ⊞ and *boxminus*⊟ operation are used to represent the updating of a matrix on Lie group by addition or subtraction with a vector on its tangent space [6]. Simplification, ⊞ or ⊟ is applied to update each element in the state vector of Equation (1) with its Lie algebra [6], yielding as x=x⊞δx.

### 2.2. Propagation Model

In order to apply standard the Extended Kalman Filter (EKF) propagation [6,36], the inertial data should be converted to a control input in a dynamic system as given:

(6) $${\dot{x}}_{\gamma }=f\left(x_{\gamma }\right)+g\left(\eta \right).$$

Note that, the input of Equation (6) is the IMU state vector of Equation (2), which only needs to process during the propagation. The focus is about how to propagate the IMU data between two adjacent camera clocks $\left[t_{\ell },t_{\ell +1}\right]$ as shown in Figure 4. Without losing generality, at a time step *t* between two IMU clocks as shown $t\in \left[t_{\gamma },t_{\gamma +1}\right]$, the first element in the IMU state vector in Equation (2) is computed as given [35]:

(7) $${}^{I_{t}}{\bar{q}}_{G}=\Phi \left(t,t_{\gamma }\right){}^{I_{\gamma }}{\bar{q}}_{G}.$$

where $\Phi \left(t,t_{\gamma }\right)$ is a transition matrix that can be solved by using Linear Time-Varying (LVI) [36].

Then, the propagation term of the rotation from time $t_{\gamma }$ to $t_{\gamma +1}$ is evaluated [7,36] as follows:

(8) $${}^{I_{\gamma +1}}{\hat{\bar{q}}}_{G}=\exp\left(\frac{1}{2}\Omega \left(\hat{\omega }\right)\Delta t\right){{}^{I_{\gamma }}\hat{\bar{q}}}_{G},$$

where $\hat{\omega }$ is assumed constantly $\hat{\omega }\left(t\right)=\hat{\omega }$ or linearly $\hat{\omega }\left(t\right)={\hat{\omega }}_{0}+\kappa t$ throughout the propagation.

To implement the algorithm in term of recursive form, the function regarding each variable in the IMU state vector of Equation (2) needs to transform into discrete-time formula according to the assumptions:

- The angular velocities between two IMU clock $t\in \left[t_{\gamma },t_{\gamma +1}\right]$ are constant $\omega_{m}\left(t\right)=\omega_{m,\gamma }.$;
- The accelerations over two IMU clock $t\in \left[t_{\gamma },t_{\gamma +1}\right]$ are constant $a_{m}\left(t\right)=a_{m,\gamma }.$

Following [7,27], the propagation of rotation in Equation (8) in discrete term yielding as:

(9) $${}_{G}^{I_{\gamma +1}}\hat{\bar{q}}=\exp\left(\frac{1}{2}\Omega \left(\omega_{m,\gamma }-{\hat{b}}_{\omega ,\gamma }\right)\Delta t\right){}_{G}^{I_{\gamma }}\hat{\bar{q}}.$$

The discrete-time position and velocity are yielded as [7,37]:

(10) $$\begin{array}{c} {}^{G}{\hat{p}}_{I_{\gamma +1}}={}^{G}{\hat{p}}_{I_{\gamma }}+{}^{G}{\hat{v}}_{I_{\gamma }}\Delta t-\frac{1}{2}{}^{G}g\Delta t^{2}+\frac{1}{2}{}^{I_{\gamma }}{{\hat{R}}_{G}}^{T}\left(a_{m,\gamma }-{\hat{b}}_{a,\gamma }\right)\Delta t^{2}\\ {}^{G}{\hat{v}}_{I_{\gamma +1}}={}^{G}{\hat{v}}_{I_{\gamma }}-{}^{G}g\Delta t+\frac{1}{2}{}^{I_{\gamma }}{{\hat{R}}_{G}}^{T}\left(a_{m,\gamma }-{\hat{b}}_{a,\gamma }\right)\Delta t \end{array}.$$

The biases specifications are maintained during the propagation as shown:

(11) $$\begin{array}{cc} \; & {\hat{b}}_{\omega ,\gamma +1}={\hat{b}}_{\omega ,\gamma }\\ \; & {\hat{b}}_{a,\gamma +1}={\hat{a}}_{a,\gamma } \end{array}.$$

According to the state vector in Equation (2), the error-state vector is established as:

(12) $${\tilde{x}}_{I}={\left[{}^{I_{\ell }}{\tilde{q}}_{G}^{T}\;\;\;\;\;{}^{G}{\tilde{p}}_{I_{\ell }}^{T}\;\;\;\;\;{}^{G}{\tilde{v}}_{I_{\ell }}^{T}\;\;\;\;\;{\tilde{b}}_{\omega_{\ell }}^{T}\;\;\;\;\;{\tilde{b}}_{a_{\ell }}^{T}\right]}^{T},$$

where $\tilde{x}$ is an error state between true state x and estimated state $\hat{x}$, as $x=\tilde{x}+\hat{x}$. The error-state vector can be written as the input of the dynamic Equation (6) as follows:

(13) $${\tilde{x}}_{I}\left(t_{\gamma +1}\right)=\Phi \left(t_{\gamma +1},t_{\gamma }\right){\tilde{x}}_{I}\left(t_{\gamma }\right)+G_{\gamma }n,$$

where $\Phi \left(t_{\gamma +1},t_{\gamma }\right)$ is a system matrix, $G_{\gamma }$ is a noise matrix, and $n={\left[n_{\omega }\;\;\;n_{a}\;\;\;n_{b\omega }\;\;\;n_{ba}\right]}^{T}$ is an inertial noise input vector. The covariance matrix [7,36] can be computed as:

(14) $$Q_{d,\gamma }=\int_{\gamma }^{\gamma +1}\Phi \left(t_{\gamma +1},\zeta \right)G\left(\zeta \right)Q_{c,\gamma }G{\left(\zeta \right)}^{T}\Phi {\left(t_{\gamma +1},\zeta \right)}^{T}d\zeta .$$

Rearrange the error states in term of dynamic Equation (13) with the input state vector of Equation (2). Finally, the covariance matrix of Equation (13) propagate from $t_{\gamma }$ to $t_{\gamma +1}$ computed as [35,36]:

(15) $$P_{\gamma +1|\gamma }=\Phi \left(t_{\gamma +1},t_{\gamma }\right)P_{\gamma |\gamma }\Phi {\left(t_{\gamma +1},t_{\gamma }\right)}^{T}+G_{\gamma }Q_{d,\gamma }G_{\gamma }^{T}.$$

with the state transition matrix $\Phi \left(t_{\gamma +1},t_{\gamma }\right)$ (A1) and the discrete time noise covariance matrix $G_{\gamma }$ (A2) as given in Appendix A. The rest of the state vector of Equation (1) does not relate to time, so their Jacobian matrices are the identity matrix. That means the state covariance matrix forms a sparse matrix, thus allowing for the exploitation of the computational saving cost. In summary, the propagation process from $t_{\gamma }$ to $t_{\gamma +1}$ is illustrated in Algorithm 1.
**Algorithm 1:** Inertial Propagation Process Algorithm
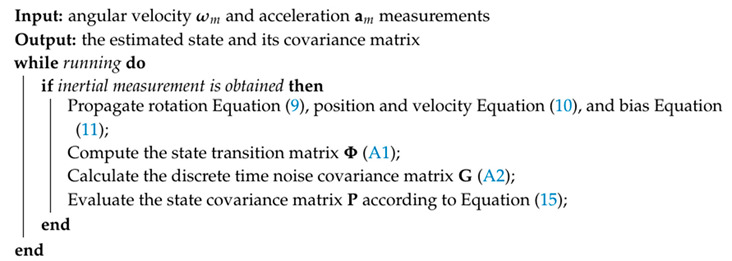


### 2.3. Visual Measurement Update Model

To use the bearing information from the camera, we first extract FAST features from the obtained images and classify them into three groups [7,27]. If a feature survives in the whole sliding window it is categorized as robust feature and if its identity is found in the state vector of Equation (4), it is called a SLAM feature. Otherwise, if the above robust feature is searched in the state vector of Equation (4) database and not detected, then it is marked by a Delay feature. Other remaining features are MSCKF features, which are generally lost track in the current camera image. Later, the delay features are used to initialize into the state vector of Equation (4) and the system covariance matrix. Finally, the SLAM and MSCKF feature are leveraged to update the state with a standard EKF process [6,36].

#### On-Manifold Correction Phase

Whenever images are acquired, the visual feature information is processed to correct the state after the inertial propagation. In order to apply standard EKF [6], all optical elements should be written in terms of the non-linear function as:

(16) $$r_{m,\ell +1}=h\left(x_{\ell +1}\right)+n_{m,\ell +1},$$

where $r_{m,\ell +1}$ is the measurement vector, $x_{\ell +1}$ is the state vector represented on manifold M, and $n_{m,\ell +1}∼N\left(0,R_{m,\ell +1}\right)$ is the white measurement noise. We linearize Equation (16) around the current estimated state ${\hat{x}}_{\ell +1|\ell }$, therefore, the current zero-mean error state is given as:

(17) $${\tilde{r}}_{m,\ell +1}=r_{m,\ell +1}-h\left({\hat{x}}_{\ell +1|\ell }\right)=H_{\ell +1}{\tilde{x}}_{\ell +1|\ell }+n_{m,\ell +1},$$

where $H_{\ell +1}$ the Jacobian matrix of the error state. From Equation (17), the standard EKF is used to update the state on manifold as follows:

(18) $${\hat{x}}_{\ell +1|\ell +1}={\hat{x}}_{\ell +1|\ell }⊞K_{\ell +1}\left(z_{m,\ell +1}-h\left({\hat{x}}_{\ell +1|\ell }\right)\right)={\hat{x}}_{\ell +1|\ell }⊞K_{\ell +1}{\tilde{r}}_{m,\ell +1}.$$

(19) $$\begin{array}{c} P_{\ell +1|\ell +1}=P_{\ell +1|\ell }-K_{\ell +1}H_{\ell +1}P_{\ell +1|\ell } \end{array}.$$

(20) $$K_{\ell +1}=P_{\ell +1|\ell }H_{\ell +1}^{T}{\left(H_{\ell +1}P_{\ell +1|\ell }H_{\ell +1}^{T}+R_{m,\ell +1}\right)}^{-1}.$$

**Figure 5:**
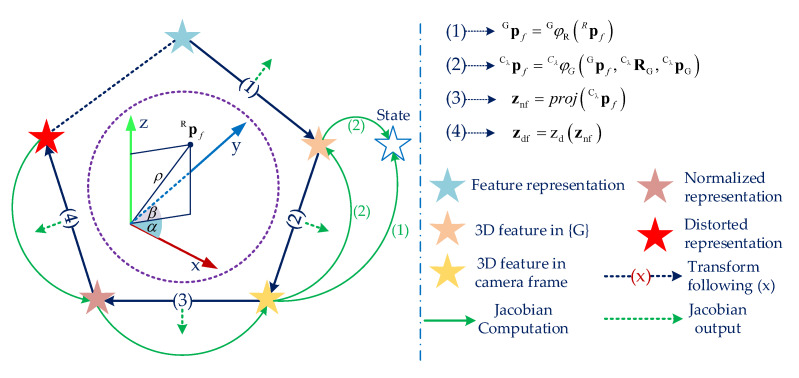
A demonstration of the transform between a 3D and 2D visual feature representation. The dark blue arrows indicate how to convert 3D feature representation to 2D data on the distorted image plane, and the green arrows show the processes to compute the Jacobian concerning a variable by using the chain rule of differentiation. Inside the brown circle presents the Anchored Inverse Depth model [7] of a visual feature.

The observed visual information is represented on the distorted image plane, therefore the measurement needs to transform 3D feature representation to 2D data in the image plane as shown in Figure 5. For a particular variable, we need to transform and calculate Jacobian based on the obtained information of the visual feature.

For example, Jacobian of a variable in state vector Equation (1) can be determined by leveraging the chain rule of differentiation, which is explained as in Figure 5, as shown:

(21) $$\frac{\partial z_{df}}{\partial x}=\frac{\partial z_{d}\left(.\right)}{\partial z_{nf}}\frac{\partial \mathrm{proj}(.)}{\partial {}^{C_{\ell }}p_{f}}\frac{\partial {}^{C_{\ell }}\varphi_{G}\left(.\right)}{\partial x}+\frac{\partial z_{d}\left(.\right)}{\partial z_{nf}}\frac{\partial \mathrm{proj}(.)}{\partial {}^{C_{\ell }}p_{f}}\frac{\partial {}^{C_{\ell }}\varphi_{G}\left(.\right)}{\partial {}^{G}p_{f}}\frac{\partial {}^{R}\varphi_{C_{\ell }}\left(.\right)}{\partial x}.$$

Then, we compute the residuals of the feature by employing the acquired measurements in the sliding window (for j=ℓ−m+1,…,ℓ+1):

(22) $$\begin{array}{ccc} {\tilde{z}}_{ij} & =z_{ij}-h_{d}\left({\hat{x}}_{\ell }{,}^{G}{\hat{p}}_{f_{i}}\right)\; & ≃H_{x_{ij}}\left({\hat{x}}_{\ell }{,}^{G}{\hat{p}}_{f_{i}}\right){\tilde{x}}_{\ell }+H_{f_{ij}}\left({\hat{x}}_{\ell }{,}^{G}{\hat{p}}_{f_{i}}\right){}^{G}{\tilde{p}}_{f_{i}}+n_{ij} \end{array},$$

where ${}^{G}{\tilde{p}}_{f_{i}}$ and ${\tilde{x}}_{\ell }$ are the error position of 3D feature and the current error state vector evaluated at the j-th measurement as shown in the vector of Equation (1), respectively. Jacobian matrices $H_{x_{ij}}$ and $H_{f_{ij}}$ are computed at $x_{ij}$ and ${\tilde{p}}_{f_{i}}$ by leveraging Equation (21). We then compact all residuals from the total observed measurements of the feature, as computed:

(23) $${\tilde{z}}_{i}≃H_{x_{i}}\left({\hat{x}}_{\ell +1|\ell }{,}^{G}{\hat{p}}_{f_{i}}\right){\tilde{x}}_{\ell +1|\ell }+H_{f_{i}}\left({\hat{x}}_{\ell +1|\ell }{,}^{G}{\hat{p}}_{f_{i}}\right){}^{G}{\tilde{p}}_{f_{i}}+n_{i},$$

where ${\tilde{z}}_{i}$, $H_{x_{i}}$, $H_{f_{i}}$, and $n_{i}$ [27] are block matrices formed by the elements in Equation (22). However, a severe problem in Equations (22) and (23) is that the primitive covariance matrices $P_{xf}$, $P_{ff}$, $P_{nf}$ related to the feature are unknown. Therefore, the linearized measurement in Equation (23) need to eliminate the error feature ${}^{G}{\tilde{p}}_{f_{i}}$. To this end, we apply QR decomposition [38] by performing the null-space, then the result of Equation (23) is yielded as [7,27]:

(24) $${\tilde{z}}_{i}≃H_{x_{i}}{\tilde{x}}_{\ell +1|\ell }+Q_{1}R_{1}{}^{G}{\tilde{p}}_{f_{i}}+n_{i}.$$

Multiplying both side of Equation (24) by $Q_{1}^{T}$, noting that $Q_{1}$ and $Q_{2}$ are orthogonal, we have:

(25) $${\tilde{z}}_{o,\ell +1}≃H_{o,\ell +1}{\tilde{x}}_{\ell +1|\ell }+n_{o,\ell +1}.$$

In addition, we can also apply the QR decomposition by using Givens rotations [38] to reduce more computational complexity, given as:

(26) $${\tilde{z}}_{n,\ell +1}≃H_{n,\ell +1}{\tilde{x}}_{\ell +1}+n_{n}.$$

The final step employs the EKF update process with Equations (25) and (26) introduced as in Equations (19) and (20), we have:

(27) $$\begin{array}{c} {\hat{x}}_{\ell +1|\ell +1}={\hat{x}}_{\ell +1|\ell }+P_{\ell +1|\ell }H_{n,\ell +1}^{T}{\left(H_{n,\ell +1}P_{\ell +1|\ell }H_{n,\ell +1}^{T}+R_{o}\right)}^{-1}{\tilde{z}}_{n,\ell +1}\\ P_{\ell +1|\ell +1}=P_{\ell +1|\ell }-P_{\ell +1|\ell }H_{n,\ell +1}^{T}{\left(H_{n,\ell +1}P_{\ell +1|\ell }H_{n,\ell +1}^{T}+R_{o}\right)}^{-1}H_{n,\ell +1}P_{\ell +1|\ell }^{T} \end{array}.$$

A large number of features detected and processed at the same time instance is the most common scenario. In order to save more computational cost, we can stack residuals of all features in Equation (26) into a block, to yield:

(28) $${\tilde{z}}^{o}≃H^{o}{\tilde{x}}_{\ell +1|\ell }+n^{o}.$$

Similarly, we employ the thin QR factorization [39] of $H^{o}=QH$ for residual ${\tilde{z}}^{o}$, given as:

(29) $$\tilde{z}=Q^{T}{\tilde{z}}^{o}≃H\tilde{x}+n.$$

Therefore, implementing the standard EKF update [6] for Equation (29) to get the final estimation result.

The last step is state augmentation of Equation (1), which occurs when obtaining a new image as in Equation (3) or inserting a new SLAM feature in Equation (4). In the first scenario, when receiving a new image, we append the latest camera pose to the state vector of camera clones in Equation (3) with a copy of the indicated pose and its covariance matrix, associated with the IMU state of Equation (2). The similar work is applied when removing a camera clone out of the sliding window. The following situation arrives when initializing a new SLAM feature *f* into the state vector Equation (4), we do the same process as presented above. Once having the covariance matrix $P_{xx}$ and cross-correlation $P_{xf}$ matrix [7,27], the augmented covariance matrix will be inserted into the feature covariance matrix, and we have:

(30) $$P^{f}=\left[\begin{array}{cc} P_{xx} & P_{xf}\\ P_{fx} & P_{ff} \end{array}\right]=\left[\begin{array}{cc} P_{xx} & P_{xf}\\ P_{xf}^{-1} & P_{ff} \end{array}\right].$$

## 3. Multi-Stage Outlier Removal in the Stochastic Environments

The accuracy of the state estimation system by using bearing information of the camera is typically influenced by dynamic objects and illumination. This section presents a multi-stage outlier removal method to overcome the problem. The generic system is presented as in Figure 2, and the overall processes steps are illustrated as:

1. *Stage 1*: The FAST features is extracted, then the KLT Feature Tracker algorithm is used to find the corresponding with RANSAC. We perform forward-backward-up-down matching to correct the tracking;
2. *Stage 2*: 3D feature triangulation with optimization solution is utilized to calculate the 3D position and the outlier rejection is also performed in this phase;
3. *Stage 3*: A robust outlier rejection scheme is proposed in which it considers the estimated state motion with the feedback information of the robot poses and velocity;
4. *Stage 4*: During the EKF update as shown in Equation (27), statistical analysis is used with the chi-squared test to carry out outliers from inliers.

Then stage 1 is used to find robust feature correspondence. An image first is divided into blocks and FAST features are extracted with multi-threading process for each image block to increase the computational speed [8,33]. Next, the optical flow with the KLT tracking algorithm with RANSAC [33,34] is employed to determine the feature corresponding in the boundary of it.

**Figure 6:**
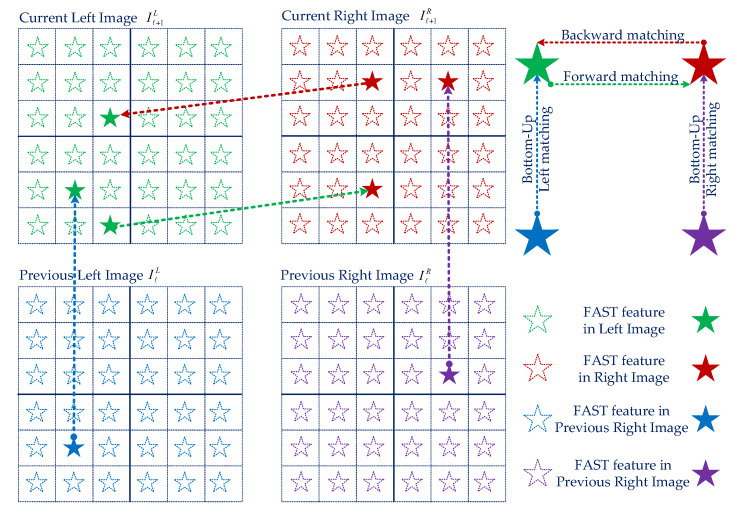
The demonstration of the proposed matching strategies for the stereo camera on the image plane using forward-backward-up-down tracking. The star shape represents a visual feature extracted by the FAST algorithm, and the arrow indicates the feature matching process.

However, the mobile robot regularly operates with sharp turn rotations, which effect the losing of optical tracking. To overcome this difficulty, we propose a robust feature matching based on the forward-backward-up-down optical flow tracking. For the current images of the left camera $I_{\ell +1}^{L}$ and the right camera $I_{\ell +1}^{R}$, we find the forward corresponding from image $I_{\ell +1}^{L}$ to image $I_{\ell +1}^{R}$, then use the backward optical flow tracking from image $I_{\ell +1}^{R}$ to $I_{\ell +1}^{L}$. Later, to avoid the illumination effect, we apply the up-down corresponding of the previous image $I_{\ell }^{L}$ to the current image $I_{\ell +1}^{L}$ of the left camera, and the previous image $I_{\ell }^{R}$ to the current image $I_{\ell +1}^{R}$ of the right camera. Stage 1 is exhibited in Figure 6.

In the next stage, when we have robust feature *f*, the triangulation is utilized to find the 3D position of the feature concerning the anchor pose. The linear triangulation [7,8,27] is applied to find the initial position of the feature. In this method, we use *m* obtained measurements of the feature and stack them into a block matrix with the form of a linear equation as follows:

(31) $$K_{2m\times 3}{}^{A}p_{f}=H_{2m\times 1},$$

where $K_{2m\times 3}$ is a 2m×3 matrix related to the bearing information with respect to the anchor position, and $H_{2m\times 1}$ is a 2m×1 matrix corresponded both to the bearing information and the transition of the feature to the anchor camera position [27]. By using the Householder rank-revealing QR decomposition [27,38] to efficiently solve with a condition number [38] of linear Equation (31), we then apply the condition number to reject outliers which sensitives to the error. After initialization, the more accurate feature position can be obtained by utilizing non-linear optimization, given as:

(32) $${}^{A}u_{f}^{*},{}^{A}v_{f}^{*},{}^{A}\rho_{f}^{*}=\underset{{}^{A}u_{f},{}^{A}v_{f},{}^{A}\rho_{f}}{\arg\min}\sum_{i=1}^{m}∥z_{f}^{i}-h_{f}^{i}\left({}^{A}u_{f},{}^{A}v_{f},{}^{A}\rho_{f}\right)∥,$$

where ${}^{A}u_{f}^{*},{}^{A}v_{f}^{*},{}^{A}\rho_{f}^{*}$ are optimal values of the 3D feature position with respect to the anchor pose, $z_{f}^{i}$ is the bearing measurement obtained in the image *i*, and $h_{f}^{i}\left(.\right)$ is a function concerning to the 3D feature position. The least-squares problem of Equation (32) is solved by the Levenberg–Marquart (LM) algorithm [8,27]. After that, the feature is filtered out if its depth is out of range or the baseline ration [8,37] is higher than a threshold.

Stage 3 is applied before utilizing the EKF update process. We focus on how to remove outliers by adopting the relation between the optical flow and the robot motion.

**Figure 7:**
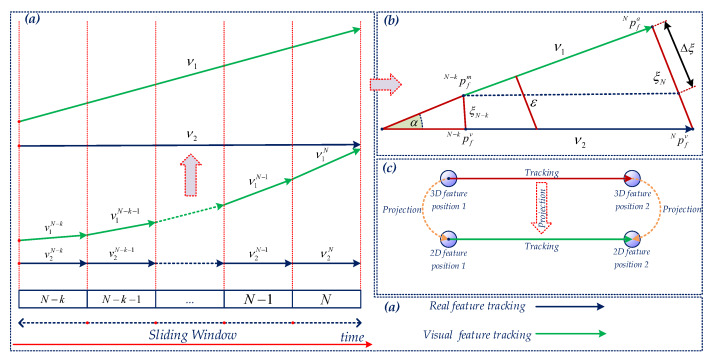
The illustration of the outlier removal strategy of a dynamic visual feature. (**a**) 2D feature tracking of the feature within a sliding window from time steps N−k to *N*, where each green arrow indicates the optical flow of the feature observed in the image plane and dark green arrow represents the estimation movement of the feature in each period. (**b**) The geometrical method based-outlier removal from start to end time step of the sliding window, the results are inferred from (**a**). (**c**) The half above represents the tracking in the 3D environment, and the half bellow demonstrates the projection of the feature on the image plane.

The ideal solution is to use the 3D feature position of each feature, then project and establish a vector that is reflected by the camera motion in the sliding window called the visual vector. Another vector is acquired from the captured bearing information of the camera named the actual vector. Once the vectors are established, they are used to compute a relation error [32], then the error is compared with a threshold to detect the outlier. The whole process is presented in Figure 7. Assume that feature *f* is needed to be considered with the estimated 3D position ${}^{A}p_{f}$. The feature position is firstly converted to the {G} coordinate. Then, we transfer it to each camera coordinate $\left\{C_{i}\right\}$ in the sliding window and project it into the normalization image plane of the camera $C_{i}$ as shown in Figure 5  and Figure 7c. We then have a set $\Omega_{1}$ of the actual vector $v_{1}^{N-i}\left(i=N-k\to N\right)$, and a group $\Omega_{2}$ of the visual vector $v_{2}^{N-i}\left(i=N-k\to N\right)$ as shown in Figure 7a. We use only the first and the last vector in set $\Omega_{1}$ and $\Omega_{2}$, then the actual vector $v_{1}$ and the visual vector $v_{2}$ are established as shown in Figure 7b. The vectors are calculated as follows:

(33) $$\begin{array}{c} v_{1}={}^{N}p_{f}^{a}-{}^{N-k}p_{f}^{a}\\ v_{2}={}^{N}p_{f}^{v}-{}^{N-k}p_{f}^{v} \end{array}.$$

With the assumption that the distances $\xi_{N-k}$ between the initial points of both vectors are small, therefore the error of endpoint is approximated as $\Delta \xi ≃\xi_{N}$. To avoid the sensitivity of visual vector $v_{2}$, we employ the normalized re-projection error ε [32], as yielding:

(34) $$\left\{\begin{array}{c} \varepsilon_{C_{L}}^{2}={∥\frac{\Delta \xi^{C_{L}}}{v_{1}^{C_{L}}}∥}^{2}\\ \varepsilon_{C_{R}}^{2}={∥\frac{\Delta \xi^{C_{R}}}{v_{1}^{C_{R}}}∥}^{2} \end{array}\right.,$$

where $\varepsilon_{C_{L}}$ and $\varepsilon_{C_{R}}$ are the normalized re-projection error of left and right camera, respectively. We finally compare the error with a threshold and remove the feature if it is higher than the limit. Nevertheless, in practice, the camera performance depends on its linear velocity and rotation. Thus, we propose a threshold which is adapted as a function of the linear and angular velocity of the robot, as yielding:

(35) τ=f(ω,v).

More simply, the update threshold is a linear combination of modular of the linear and angular velocity, as shown:

(36) $$\tau =f\left(\omega ,v\right)=K_{\omega }{∥\omega ∥}^{2}+K_{v}{∥v∥}^{2}.$$

where the parameters $K_{\omega }$ and $K_{v}$ are selected depending on the camera.

Finally, stage 4 is studied when the computed results are not close enough to the expected solution, which can influence the outcome of the estimation algorithm. To this end, the Mahalanobis gating test [8,27] is applied. After leveraging the null-space projection in Equation (25), the linearization results give us ${\tilde{z}}_{o,\ell +1}$ and $H_{o,\ell +1}$. In this case, we need to find the difference between the observation and estimation values. The residual covariance is given as $H_{0,\ell +1}P_{\ell +1,\ell }H_{0,\ell +1}^{T}$, then the condition value is computed as follows:

(37) $$\xi_{i}={\left({\tilde{z}}_{0,\ell +1}\right)}^{T}{\left(H_{0,\ell +1}P_{\ell +1,\ell }H_{0,\ell +1}^{T}+\sigma^{2}I\right)}^{-1}\left({\tilde{z}}_{0,\ell +1}\right).$$

Once the value $\xi_{i}$ is computed, it is used to compare with a threshold given by the Chi-squared distribution $\chi^{2}$ of the 95-th percentile cumulative density function. Here the degrees of freedom of the $\chi^{2}$ is the number of rows in the residual vector ${\tilde{z}}_{o,\ell +1}$. If the value $\xi_{i}$ is larger than the threshold, then remove the feature from the inlier. The Chi-squared distribution $\chi^{2}$ can be calculated at this stage. However, to save the computational cost, we apply an efficient look-up table to search the value.

## 4. Experimental Results

In this section, the details of experimental results are presented and compared with the state-of-the-art methods on public datasets. Moreover, the proposed algorithm is evaluated within a sensor platform in indoor stochastic environments. First, we develop the proposed methodology, as shown in Algorithm 2. Herein, the number of the sliding window is fixed to (K=5). The maximum number of extracted FAST features is set to 400, and the maximum value of the SLAM feature in the state vector is limited to 50 (ρ=50). The proposed algorithm runs in real-time based on the laptop computer Legion Y520 with device specification as an Intel Core i7-7700HQ four-core 2.8 GHz processors, GTX 1060 GPU, and 16 GB RAM. However, this algorithm only utilizes CPU to evaluate results. We develop and perform the algorithm on top of Ubuntu version 16.04.5 LTS with the Robot Operating System (ROS)  version-Kinetic [40] written in C/C++.    
**Algorithm 2:** A Robust Stereo VINS Algorithm Based on Multi-Stage Outlier Removal
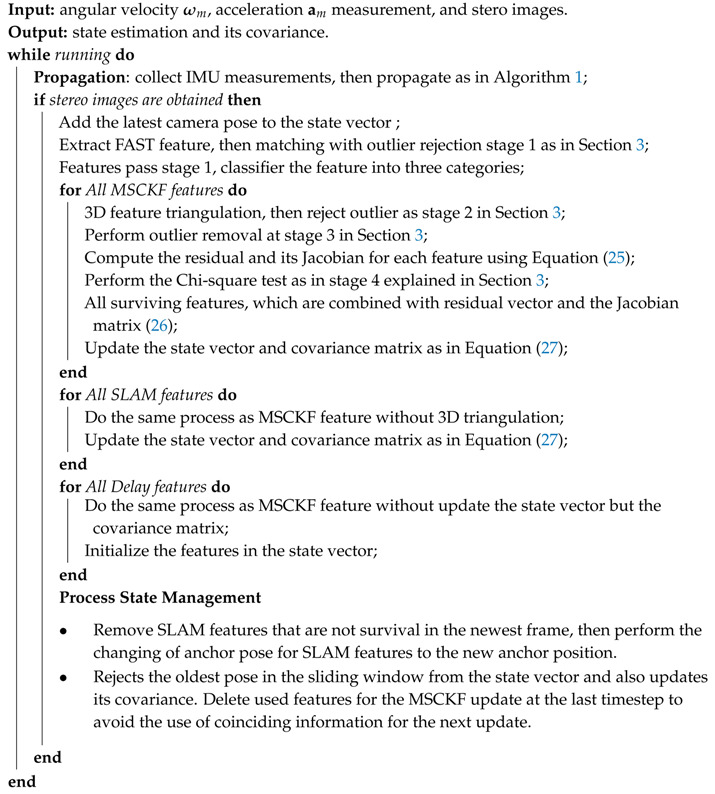


### 4.1. Experimental Results on the Public Datasets

In this experiment, the visual-inertial EuRoC dataset [41] with 11 sequences is utilized. The datasets were collected by on-board a Micro Aerial Vehicle (MAV) in three categories of static indoor environments. The available data consists of 20 Hz stereo images and 200 Hz of MEMS IMU ADIS16448. The camera intrinsic, camera-IMU extrinsic, and spatio-temporal calibration parameters are given. These datasets also support 200 Hz ground-truth, which is evaluated by using the 3D and 6D motion capture systems consist of the Vicon, Leica MS50 laser tracker, and Leica MS50. We evaluate the proposed algorithm without using SLAM landmarks supported by the EuRoC dataset. Our algorithm can operate successfully in real-time for all sequences of the datasets. We also conduct the comparison with VIO algorithms as follows:

1. (Stereo) S-MSCKF VIO [8]: A state-of-the-art filtering-based method with open-sourced, which implemented from initial version MSCKF [7], uses stereo images for high accuracy navigation;
2. VINS-Fusion VIO [13]: A modern optimization-based solution is an extended version of VINS-mono [14]. This method utilizes the IMU pre-integration with a factor-graph optimization. The loop closure is disabled for this test;
3. Basalt VIO [15]: The most recent state-of-the-art open-sourced VIO, which employs the fix-lag smoother based-optimization methodology. This method uses stereo key-frame to extract the relevant information for VIO based on the nonlinear factor recovery.

**Figure 8:**
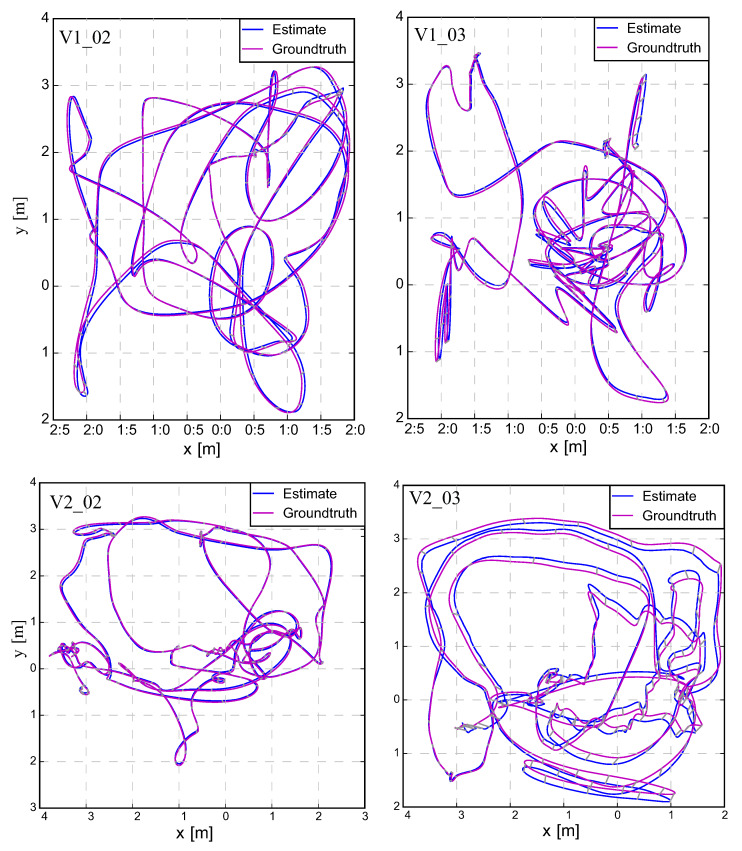
A comparison between the estimated trajectory (blue line) and the ground-truth (magenta line) by the proposed method on sequence V1_02, V1_03, V2_02, and V2_03 are expressed following the gravity direction.

**Figure 9:**
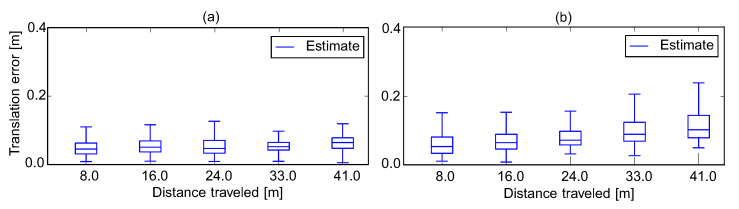
The comparison of the relative translation error of Basalt and our method on sequence V2_02. (**a**) The relative translation error of our proposed method and (**b**) the relative translation error of Basalt.

**Figure 10:**
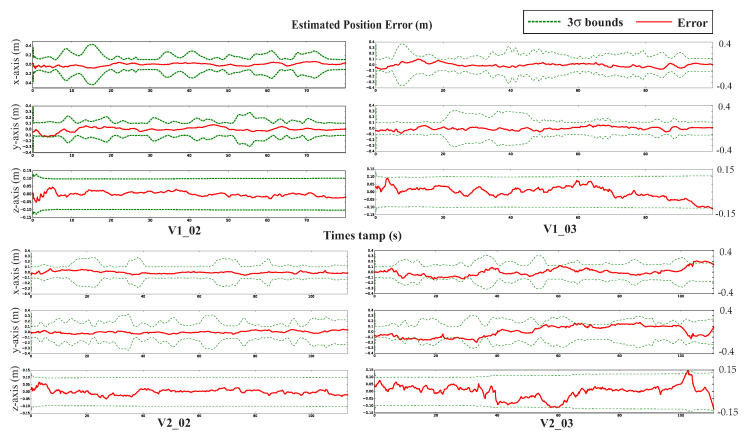
State estimation errors (red-line) and 3 σ bounds (green-dashed line) for the proposed method in the EuROC dataset with sequences V1_02, V1_03, V2_02, and V2_03.

To perform the comparison, we use the SLAM VIO trajectory evaluation toolbox [42] to compute the Root Mean Square Errors (RMSE) and Absolute Trajectory Error (ATE) for each sequence from EuRoC datasets, the experimental outcomes are shown in Table 1. Our proposed results outperform S-MSCKF and VINS-Fusion in the most sequences of the EuRoC dataset, and slightly less than Basalt overall. Table 1 indicates that S-MSCKF filtering-based VIO had the worst result, followed by the VINS-Fusion based-optimization method. Although the Basalt solution was better than ours in the case of V1_01, MH_04, and MH_05, the proposed approach outperformed in sequences V1_03 and V2_02. Other routes had similar results. The best cases of our proposed method were deployed in the V1_02, V1_03, V2_02, and V2_03 sequence, which are illustrated in Figure 8. Figure 9 provides an example of a comparison of the relative transition error between our method and the Basalt V2_02 sequence. Furthermore, the three-sigma bounds of the state estimation errors were considered [27], as shown in Figure 10. The boundaries with ±3σ reported that more than 99% error fell in the confidence regions. This bounded uncertainty outcomes indicated that the proposed system was a well-functioning and robust filter.

We examined the processing time of each task provided in Table 2. Our algorithm was implemented and operated in parallel on CPU resources. Table 2 shown that the most consumption time was the tracking process with feature extraction and feature matching. In contrast, the shortest processing period was propagation, which was less than a half millisecond. The time consumed for the feature update and initialization increased linearly on the number of features to be updated [7]. In summary, the total processing time was about 26 ms, approximately equal compared with 25 ms of S-MSCKF. The optimization-based VIO methods consumed near double-time compared with our process. In detail, the VINS-Fusion and Basalt use factor-graph optimization spent 60 ms and 54 ms, respectively, compared with our proposed method which only took 26 ms. The quantitative results show that the proposed method had a similar accuracy but outperformed the state-of-the-art VIO techniques with only a half computational complexity.

### 4.2. Experimental Results in an Indoor Dynamic Environments

**Figure 11:**
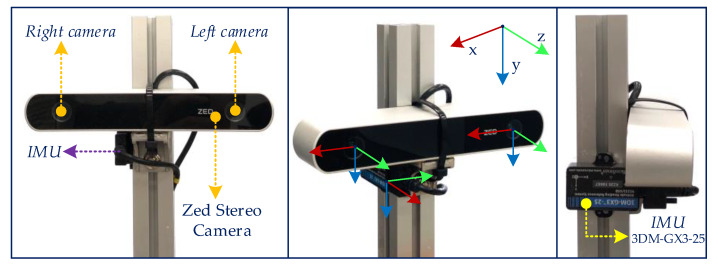
The hardware setup consists of a stereo Zed camera and an inertial sensor 3DM-GX3-25.

In order to evaluate the experimental results in the indoor dynamic environments, we built a sensor system comprised of a Zed stereo camera and an IMU 3DM-GX3-25, which are rigidly attached on a convenient light-weight platform as shown in Figure 11.

During an evaluation of the experiment, the stereo camera’s frequency was set at 30 Hz with an image resolution that captured 640×480 while the IMU signal was sampled at a rate of 200 Hz.

**Figure 12:**
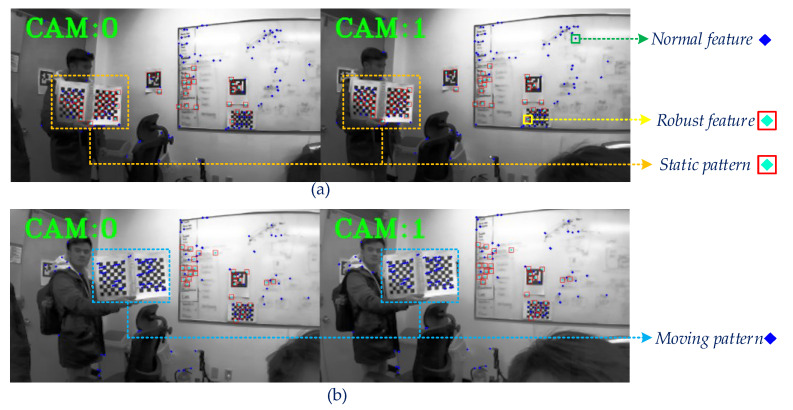
Screen snapshots using the Robot Operating System (ROS) image toolbox during the indoor lab test with a dynamic pattern. (**a**) In this case, the pattern is static and many features identified the robust feature. (**b**) When the pattern begins moving, the robust feature turns to the normal feature.

Our results could detect and remove outlier efficiently, as shown in Figure 1 and Figure 12. The use of multi-stage outlier removal could efficiently discard the dynamic features, indicated by the blue circles in Figure 12b.

**Figure 13:**
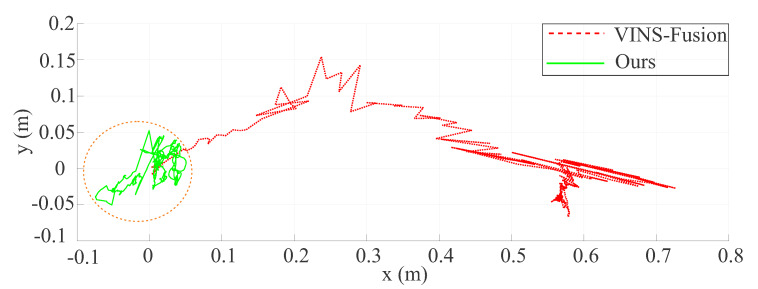
The estimated trajectory (blue line) drifts over time compared with VINS-Fusion [13] (red dash line) in a very high dynamic environment.

We first evaluated the proposed algorithm inside a high-dynamic room where the people are continuously moving in the scope of a static camera. The RMSE of the proposed method was only 0.02 compared with the drift of VINS-Fusion [13] which was more than half a meter, as shown in Figure 13.

**Figure 14:**
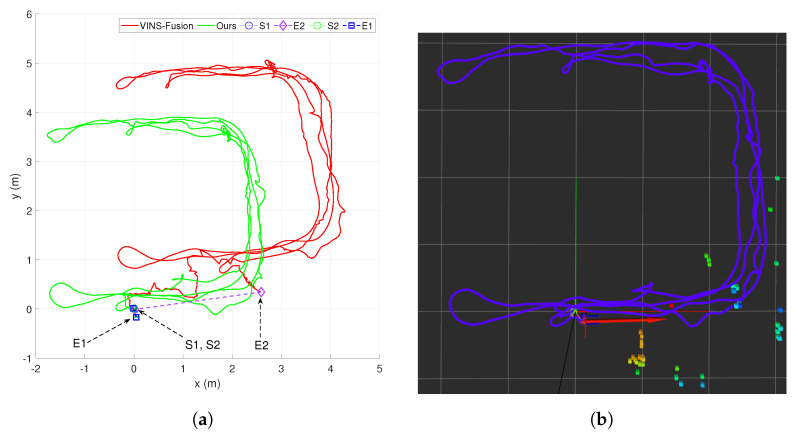
The comparison between the proposed method and VINS-Fusion [13] when moving inside a lab room around 6 × 6 m^2^ with a dynamic person moving ahead. (**a**) The comparison between the proposed method and VINS-Fusion [13]. (**b**) The visualization of our method trajectory shown in Rviz.

In the second situation, the sensor system was moved around a small lab room of around 6×6 m${}^{2}$ while a pedestrian with a pattern walked inside the scope of the camera. We evaluated the position precision by applying KITTI standard [43], which is calculated by the ratio of the drift when returning to the initial pose and the total length of the trajectory. The rotation accuracy was also the relative of error bearing to the entire path. The result of the traveling path is illustrated in Figure 14, note that the sensor system turned around many times in this case. After getting the last pose, we computed the position precision $\Delta p=\frac{0.2563}{45.52}=0.56$ (cm/m), and the bearing accuracy was $\Delta \varphi =\frac{2.4443}{45.52}=0.0541$ (deg/m). Although the system was employed to do several sharp turn rotations, the result still achieved a high precision. VINS-Fusion [13] was also used to perform this experiment however, the accuracy was about 4 (cm/m), which is approximately 10 times less than our algorithm as shown in Figure 14.

**Figure 15:**
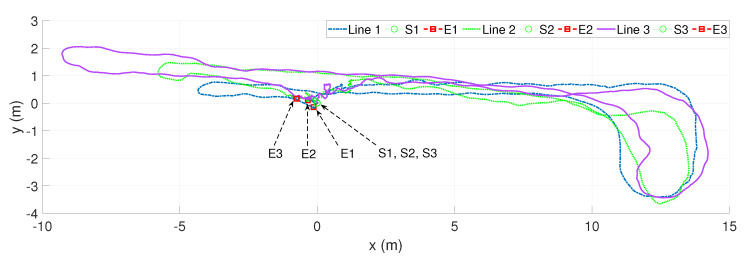
The estimated trajectories in a stochastic environment along the hallway outside our lab around 7m×25m with various scenarios are illustrated. Line 1 is a path without the dynamic object; Line 2 is with the moving person but sparse, Line 3 is with a very dense active person; all cases are under the influence of illumination. Point S1, S2, and S3 are start poses of all paths while E1, E2, and E3 are end positions of scenario 1, 2, and 3, respectively.

**Figure 16:**
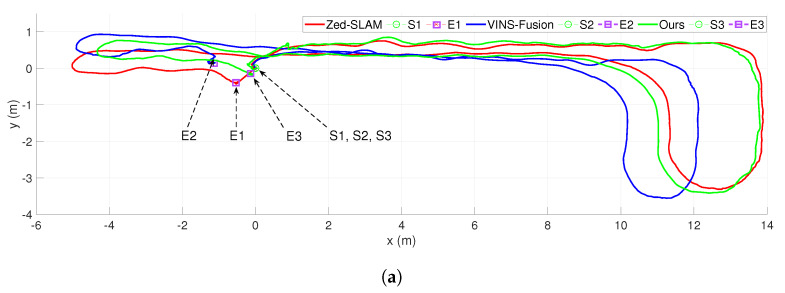

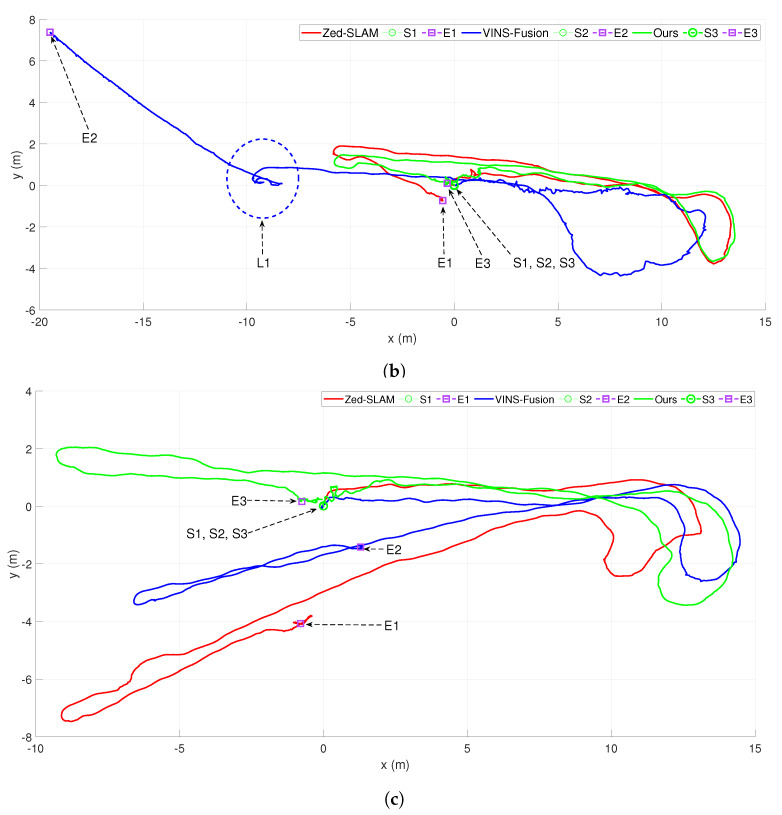
The comparison of estimated trajectories between our proposed method with VINS-Fusion and Zed SLAM in various situations, as described in Figure 15. (**a**) The result in scenario 1. (**b**) The result in scenario 2, where L1 lost the tracking area. (**c**) The result in scenario 3.

For the last scenario, we conducted the sensor configuration moving along a hallway of around 7m×25m and returned to the initial position. The result is presented in Figure 15. Herein, Line 1 (case 1) shows the estimated path, which is measured by moving the sensor system along the hallway without a dynamic object but was effected by the illumination of electric lights. In case 2, a person moves along with a pattern and appears sparse in the camera’s scope. In this scenario, the dot green line (Line 2) presents the estimated trajectory inside this environment under dynamic and illumination effects. Line 3 (case 3), as shown in Figure 15, demonstrates the navigation path in a high dynamic and illumination environment. In this case, a person with a pattern moving with high frequency inside the scope of both cameras. Figure 16 demonstrates the comparisons between our method, VINS-Fusion [13], and Zed SLAM [44] supported by the producer. Figure 16a depicts our proposed method’s results and the Zed SLAM algorithms, while VINS-Fusion gained a poor accuracy with an approximate 1 m drift. In particular, Zed SLAM could detect a loop closure at the endpoint, then adjusted the end pose to coincide with the initial position. Nevertheless, if we do not consider the loop closure detection, the proposed algorithm gets a better result. As observed from Figure 16b, although adding loop closure technical, our method with an error of 0.78 outperformed the Zed SLAM’s result of 1.975. VINS-Fusion lost tracking in area $L_{1}$, so it gained a severe outcome of around 47 (cm/m). The worst of Zed SLAM is shown in Figure 16c, which had a wrong direction when returning to the initial pose while our solution had a stable result with only an error of 1.4%. VINS-Fusion also failed when coming back to the original position, but its accuracy was approximately twice better than Zed SLAM. The position and bearing accuracy of our method and the comparison are presented in Table 3.

## 5. Conclusions

The problem of vision-aided inertial navigation has achieved significant progress over a decade. Nevertheless, stochastic environments typically consist of dynamic objects and illumination invariance. Thus the precision and robustness of the estimation system is adversely affected. In this paper, we studied a robust visual-inertial navigation system based on the tightly-coupled methodology within the MSCKF framework using a stereo camera and an IMU for the stochastic surroundings. The system was integrated with the multi-stage outlier removal algorithm to limit the influences of dynamic environments. Herein, visual data was applied to extract FAST features, then leveraged for the removal algorithm to reject uncertainty features. Lastly, the surviving features were employed in the correction phase of EKF. We evaluated the algorithm in the EuRoC dataset and got noticeable results compared with the state-of-the-art VINS. In addition, a sensor system consisting of a Zed camera and an inertial measurement unit was installed to examine the performance of the proposed method in the dynamic surroundings. The outcomes demonstrated that our solution outperformed the Zed SLAM and VINS-Fusion with a precision of only 0.4% to 1% depending on each scenario. The proposed estimator operated in real-time, with only 26 ms of time consumption without a GPU. Finally, this solution could enable us to obtain high-precision pose estimation in real-time under resourced-constraint devices.

## Figures and Tables

**Table 1:** Root Mean Square Errors (RMSE) of absolute trajectory error for S-MSCKF, VINS-Fusion, and Basalt compared with our algorithm on the EuRoC dataset. The bold text results are shown to be the best and X is fail to test.

Sequence	Length (m)	S-MSCKF (m)	VINS-Fusion (m)	Basalt (m)	Ours (m)
V1_01_easy	58.6	0.06	0.10	**0.04**	0.06
V1_02_medium	75.9	0.16	0.10	**0.05**	**0.05**
V1_03_difficult	79.0	0.28	0.11	0.10	**0.05**
V2_01_easy	36.5	0.07	0.12	**0.04**	0.05
V2_02_medium	83.2	0.15	0.10	0.07	**0.03**
V2_03_difficult	86.1	0.37	0.27	X	**0.13**
MH_01_easy	80.6	0.23	0.24	0.09	**0.08**
MH_02_easy	73.5	0.23	0.18	**0.06**	**0.06**
MH_03_medium	130.9	0.20	0.23	**0.07**	0.09
MH_04_difficult	91.7	0.35	0.39	**0.13**	0.18
MH_05_difficult	97.6	0.21	0.19	**0.11**	0.19

**Table 2:** A half table above indicates the processing time in milliseconds (ms) for each task in our method. The comparison with Vision-Aided Inertial Odometry (VIO) technicals is presented in a half table below. The numbers in the first column, such as 32/21/5, indicated 32 standard features, 21 SLAM features, and five Delay features treated. the Marg represents the marginalization process.

Tasks	Tracking (ms)	Propagation (ms)	Feat Update (ms)	Init and Update (ms)	Marg (ms)	Total (ms)
37/2/1	16.5	0.3	2.2	1.0	0.8	20.8
32/21/5	13.9	0.3	1.0	6.2	1.0	22.4
48/39/5	11.4	0.4	2.8	13	1.3	29.0
**Methods**	**S-MSCKF**		**VINS-Fusion**	**Basalt**	**Ours**	
Average (ms)	25		60	53	26	

**Table 3:** The comparison of the proposed method to Zed-SLAM and VINS-Fusion.

Sequences	Case 1–Line 1	Case 2–Line 2	Case 3–Line 3
Length (m)	43.70	46.69	55.02
Position error (cm/m)	0.568	0.780	1.416
Bearing error (deg/m)	0.376	0.064	0.915
Zed error (cm/m)	1.542	1.975	8.888
VINS-Fusion error (cm/m)	2.611	X (47.672)	4.384

## Appendix A. The State Transition and the Discrete Time Noise Covariance Matrix

(A1) $$\Phi \left(t_{\gamma +1},t_{\gamma }\right)=\left[\begin{array}{ccccc} {}^{I_{\gamma +1}}{\hat{R}}_{I_{\gamma }} & 0_{3} & 0_{3} & -{}^{I_{\gamma +1}}{\hat{R}}_{I_{\gamma }}J_{r}\left({}^{I_{\gamma +1}}\theta_{I_{\gamma }}\right)\Delta t & 0_{3}\\ -\frac{1}{2}{}^{I_{\gamma }}{\hat{R}}_{G}^{T}\left\lfloor {\hat{a}}_{\gamma }\Delta t^{2}\times \right\rfloor & I_{3} & \Delta tI_{3} & 0_{3} & -\frac{1}{2}{}^{I_{\gamma }}{\hat{R}}_{G}^{T}\Delta t^{2}\\ -{}^{I_{\gamma }}{\hat{R}}_{G}^{T}\left\lfloor {\hat{a}}_{\gamma }\Delta t\times \right\rfloor & 0_{3} & I_{3} & 0_{3} & -{}^{I_{\gamma }}{\hat{R}}_{G}^{T}\Delta t^{2}\\ 0_{3} & 0_{3} & 0_{3} & I_{3} & 0_{3}\\ 0_{3} & 0_{3} & 0_{3} & 0_{3} & I_{3} \end{array}\right].$$

(A2) $$G_{\gamma }=\left[\begin{array}{cccc} -{}^{I_{\gamma +1}}{\hat{R}}_{I_{\gamma }}J_{r}\left({}^{I_{\gamma +1}}{\hat{\theta }}_{I_{\gamma }}\right)\Delta t & 0_{3} & 0_{3} & 0_{3}\\ 0_{3} & -\frac{1}{2}{}^{I_{\gamma }}{\hat{R}}_{G}^{T}\Delta t^{2} & 0_{3} & 0_{3}\\ 0_{3} & -{}^{I_{\gamma }}{\hat{R}}_{G}^{T}\Delta t & 0_{3} & 0_{3}\\ \begin{array}{c} 0_{3}\\ 0_{3} \end{array} & \begin{array}{c} 0_{3}\\ 0_{3} \end{array} & \begin{array}{c} \Delta tI_{3}\\ 0_{3} \end{array} & \begin{array}{c} 0_{3}\\ \Delta tI_{3} \end{array} \end{array}\right].$$
